# Inhibition of the expression of *rgs‐3* alleviates propofol‐induced decline in learning and memory in *Caenorhabditis elegans*

**DOI:** 10.1111/cns.14004

**Published:** 2022-10-25

**Authors:** Ayang Zhao, Hongjiang Jin, Guibo Fan, Yan Li, Chenglong Li, Qi Li, Xiaofei Ma, Tianyang Zhao, Siqi Sun, Shuai Liu, Yueyue Gao, Sihua Qi

**Affiliations:** ^1^ Department of Anesthesiology The Fourth Affiliated Hospital of Harbin Medical University Harbin China; ^2^ Department of ICU The Fourth Affiliated Hospital of Harbin Medical University Harbin China

**Keywords:** learning, memory, neurodevelopment, propofol, regulators of G protein signaling

## Abstract

**Background:**

Exposure to anesthesia leads to extensive neurodegeneration and long‐term cognitive deficits in the developing brain. *Caenorhabditis elegans* also shows persistent behavioral changes during development after exposure to anesthetics. Clinical and rodent studies have confirmed that altered expression of the regulators of G protein signaling (RGS) in the nervous system is a factor contributing to neurodegenerative and psychological diseases. Evidence from preclinical studies has suggested that RGS controls drug‐induced plasticity, including morphine tolerance and addiction. This study aimed to observe the effect of propofol exposure in the neurodevelopmental stage on learning and memory in the L4 stage and to study whether this effect is related to changes in *rgs‐3* expression.

**Methods:**

*Caenorhabditis elegans* were exposed to propofol at the L1 stage, and learning and memory abilities were observed at the L4 stage. The expression of *rgs‐3* and the nuclear distribution of EGL‐4 were determined to study the relevant mechanisms. Finally, RNA interference was performed on *rgs‐3*‐expressing cells after propofol exposure. Then, we observed their learning and memory abilities.

**Results:**

Propofol time‐ and dose‐dependently impaired the learning capacity. Propofol induced a decline in non‐associative and associative long‐term memory, *rgs‐3* upregulation, and a failure of nuclear accumulation of EGL‐4/PKG in AWC neurons. Inhibition of *rgs‐3* could alleviate the propofol‐induced changes.

**Conclusion:**

Inhibition of the expression of *rgs‐3* alleviated propofol‐induced learning and memory deficits in *Caenorhabditis elegans*.

## Graphical abstract

It is the first time that intravenous anesthetics have an effect on learning and memory function in invertebrates, and a new potential mechanism has been found.

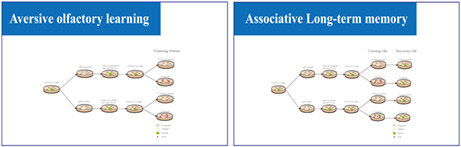

## 1 INTRODUCTION

Propofol is the most common anesthetic in clinic, which is used for induction and maintenance of anesthesia and sedation during diagnosis and treatment in children. It has many pharmacologic advantages agents such as rapid effect, short action, and fewer side effects such as postoperative nausea, comparing with other anesthetics.^1^ FDA approved the use of propofol in the maintenance of anesthesia for children born more than 2 months and the induction of anesthesia for children over 3 years old.^2^ At the same time, about 1%–2% of pregnant women used propofol during delivery.^1^ These may be premature exposure of the newborn to propofol.

The developing brain is sensitive to a variety of chemicals such as heavy metals,^3^ formaldehyde,^4^ and even PM2.5.^5^ Even minimal exposure, which is typically considered safe, could be harmful. In the developing brain, exposure to anesthesia leads to extensive neurodegeneration and long‐term cognitive deficits.^6^ *Caenorhabditis elegans* also shows persistent behavioral changes during development after exposure to anesthetics. Gentry et al.^7^ showed that larvae exposed to anesthetics administered via inhalation show deficits in chemotaxis during adult. This anomaly was observed only in nematodes exposed during the L1 developmental stage. Connor et al.^8^ demonstrated that altered locomotive behavior dynamics have a persistently effect on interneuron dynamics and circuit function in *C. elegans* after exposure to isoflurane during development.

However, the mechanism underlying nervous system damage induced by anesthetics in developing organisms remains unclear. At the cellular level, anesthesia exposure can induce structural and functional changes in cortical pyramidal neurons, including alterations in the density and dynamics of postsynaptic dendritic spines, dendritic arborization, neuronal activity, and synaptic protein expression.^9^, ^10^, ^11^ The G protein is one of the main synaptic proteins regulated by regulators of G protein signaling (RGS).^12^ RGSs are multifunctional proteins expressed in the peripheral and neuronal cells.^13^ Clinical and rodent studies have confirmed that altered expression of RGS in the nervous system contributes to the development of neurodegenerative and psychological diseases.^14^, ^15^, ^16^ Evidence from preclinical work suggests that RGSs control drug‐induced plasticity, including morphine tolerance and addiction.^13^, ^17^, ^18^

In *C. elegans*, RGS‐3 inhibits Gα activity.^19^ Gαq signal activation enhances memory consolidation and slows cognitive decline.^20^ Moreover, *rgs‐3* mutant animals can better translocate cGMP‐dependent protein kinase G (EGL‐4/PKG) in AWC neurons induced by odor.^21^ The nuclear accumulation of EGL‐4 is essential for AWC neuronal plasticity during learning.^22^ Therefore, we hypothesized that RGS‐3 inhibits the learning and memory functions of nematodes. Although studies have reported that opioids affect RGS expression, the effect of propofol on RGS expression is not known.

Here, we first aimed to examine whether exposure to propofol during brain development induced a decline in learning and memory in *C. elegans*. We then aimed to examine the expression of *rgs‐3* and the nuclear accumulation of EGL‐4 and whether the inhibition of *rgs‐3* could alleviate propofol‐induced learning and memory decrease.

## 2 MATERIALS AND METHODS

Invertebrate research, such as that involving *C. elegans*, is exempt from institutional Animal Care and Use Committee reviews under the provisions of the Animal Welfare Act and the Health Research Extension Act. All experiments were performed at the Fourth Affiliated Hospital of Harbin Medical University, TOF‐PET/CT/MR Center.

### 2.1 *C. elegans* strains

Worms were grown at 20–22°C on nematode growth medium (NGM) plates seeded with *Escherichia coli* OP50 before use. A Bristol (N2) hermaphrodite strain was used as the wild type. Imaging experiments were performed using the transgenic strains TJ356 (zIs356 [daf‐16p::daf‐16a/b::GFP + rol‐6(su1006)]) and JZ500(pyIs500 [ofm‐1p::GFP + odr‐1p::DsRed + odr‐3p::GFP::egl‐4]). N2, TJ356, and JZ500 cells were obtained from the Caenorhabditis Genetics Center (University of Minnesota, Minneapolis, MN, USA).

### 2.2 Synchronization and exposure to anesthesia

Propofol (100 mM; 2,6‐diisopropyl phenol; Macklin, China) was prepared in 10^−3^ DMSO (Macklin, China) and stored at −20°C, which was freshly prepared every day.

We used a standard protocol to bleach hermaphrodite pregnant worms and synchronize their populations. Eggs were incubated at 20°C for approximately 15 h until all eggs hatched into the first stage (L1) larvae.^23^ Transferred the larvae to Propofol‐seed or Control‐seed (10^−3^ DMSO) NGM plates for a period of time. Then, we washed them off the plates with M9 buffer, washed them again thrice to discard propofol or DMSO, and transferred the washed animals to OP50 bacteria again. After 48 h, when the worms reached the L4 stage, the training was initiated.

To avoid the influence of subjective factors on the results of this study, the experimental grouping and drug exposure were carried out by specific personnel, and the grouping of nematodes was not published before the observations were completed.

### 2.3 Aversive olfactory training

**FIGURE 1:**
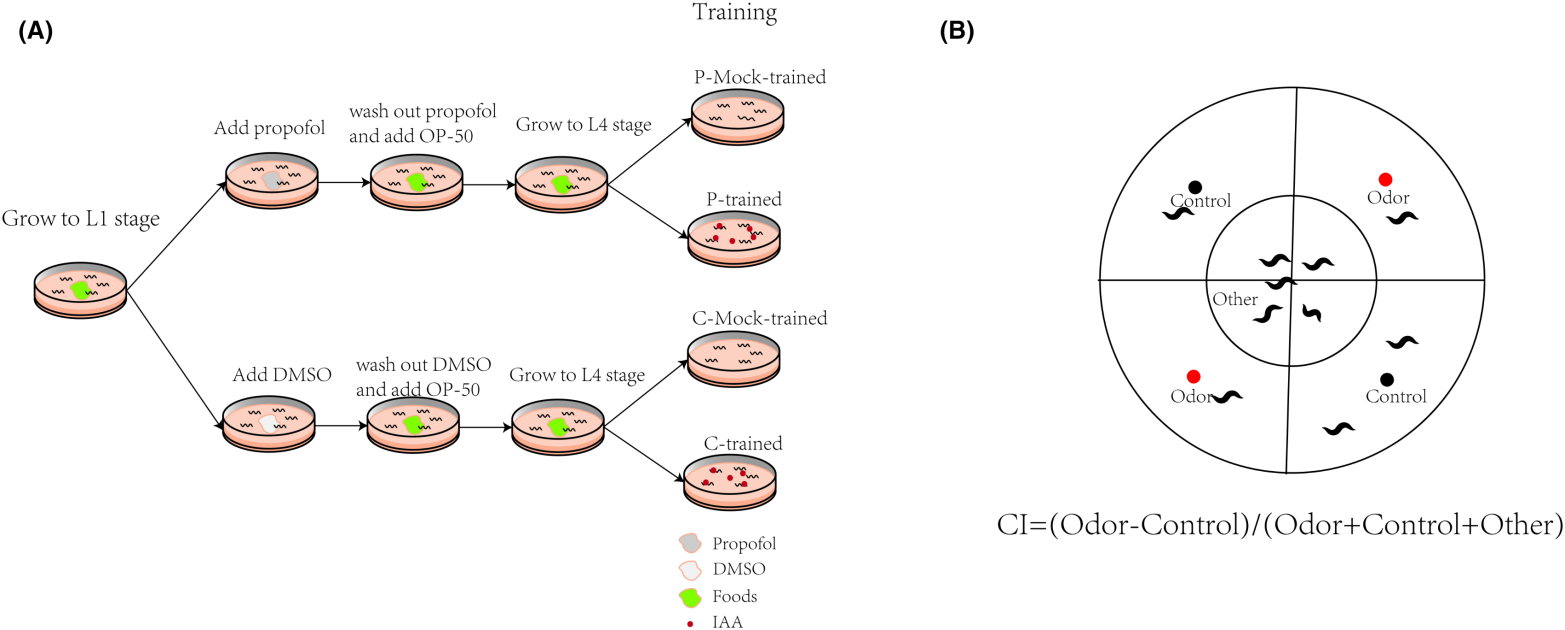
Experiment flow chart. (A) Schematic of aversive olfactory learning assay. (B) Schematic diagram of chemotaxis plate.

First, we removed the bacterial residues and transferred the worms to NGM plates with a diameter of 90 mm without access to food. The worms were divided into groups of trained animals (presence of odorant) and mock‐trained animals (absence of odorant) according to the presence or absence of an odorant during nematode starvation. We used 10^−3^ isoamyl alcohol (IAA) (Aladdin, China) as an odorant. The odorant was introduced by placing nine evenly spaced 5 μl drops on the inner surface of the cover. This process was referred to as training. After the training, we transferred the worms back to NGM plates with OP50 and challenged them (Figure 1A).^23^ Here, we refer to the process of re‐exposing the worm to the odor as challenging the animal.

### 2.4 Long‐term memory

The method was similar to that of aversive olfactory training, but the starvation duration was fixed at 24 h. After training, the worms were transferred back to NGM plates with OP50 for a period before challenging them.

### 2.5 Behavioral analyses

**FIGURE 2:**
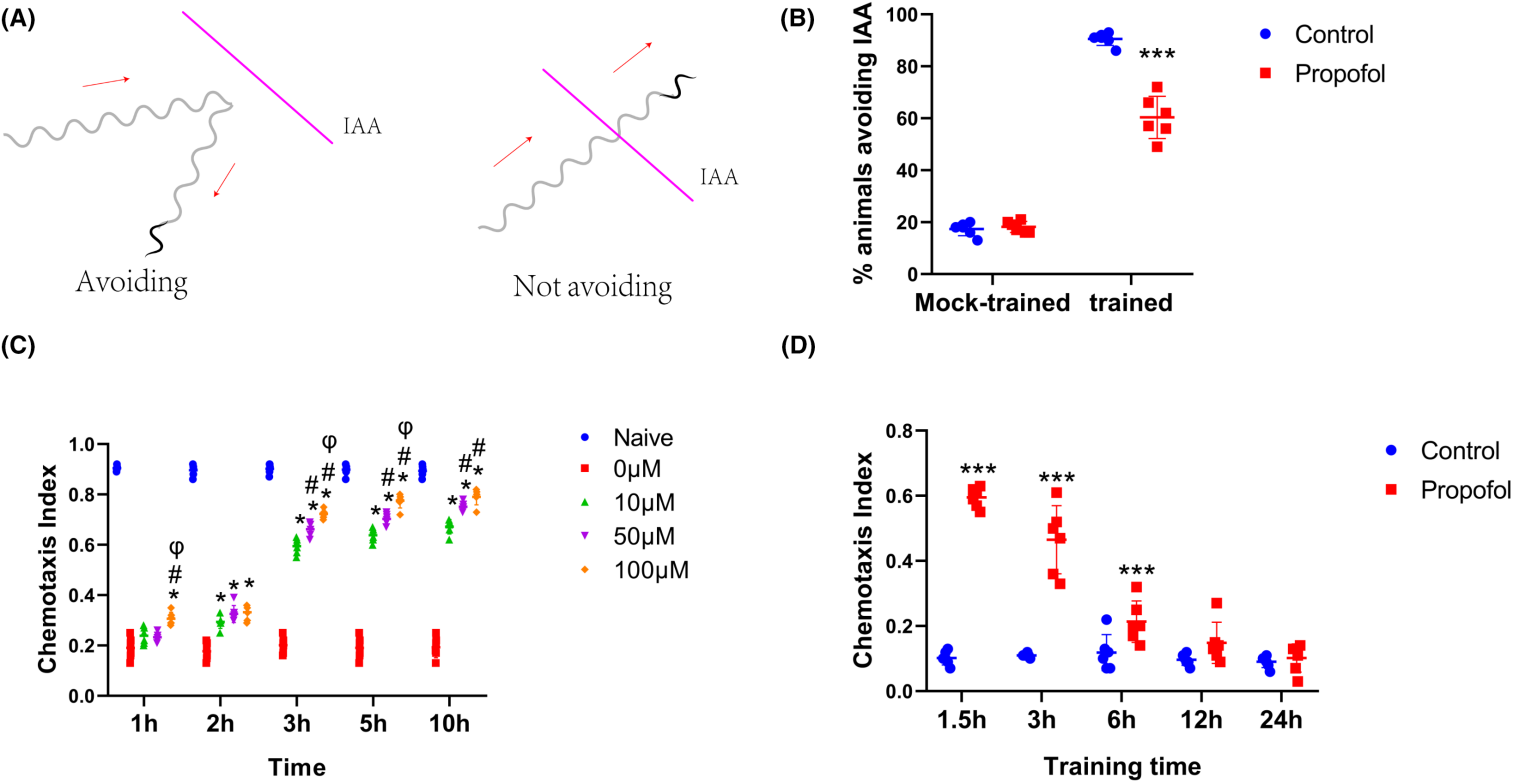
Propofol induces a decline in learning in a time‐ and dose‐dependent manner. (A) Travel paths corresponding to two different behaviors observed upon exposure to IAA. (B) Percentage of animals avoiding IAA. (C) *C. elegans* was exposed to propofol at concentrations of 0, 10, 50, and 100 μM for 1, 2, 3, 5, and 10 h. Naive represents untrained wild‐type nematodes. (D) Chemotaxis index of nematodes exposed to IAA calculated for different training intervals. *p* values were generated by one‐way analysis of variance (ANOVA). *n* = 40–60 animals per condition. The experiments were repeated six times. *p < 0 0.05 versus 0 μM, ^#^ *p* < 0.05 versus 10 μM group; ^φ^ *p* < 0 0.05 versus 50 μM group; ***p < 0 0.001 versus Control.

We picked individually trained animals, placed them on a new unseeded plate, and allowed them to adjust for 2 min. When the worm started running forward for a long time, we used a hair strand pre‐dipped in the smelling solution (10^−3^ IAA) to spread a smelling stripe vertically in front of its forward trajectory. If the worm stopped next to the extended stripe and retreated within 3 s, the behavior was termed “avoiding.” Worms that stopped briefly or continued to run forward through the stripes were rated as those who “did not avoid” (Figure 2A).

### 2.6 Chemotaxis choice assays

Chemotaxis was tested on a plate containing 2% agar poured the day before the trial. Adult worms were washed thrice with M9 buffer, and 100–200 animals were placed in the center of the plate. A total of 1 ml of IAA diluted at 1:1000 in ethanol or ethanol control was dropped on the red or black spots of the plate, and 1 ml of 1 M NaN_3_ was dropped at the same point to fix the animals arriving at the IAA or ethanol source. The test was performed on each side of the plate by counting the animals leaving the starting point after 1 h. The chemotaxis index (CI) was calculated as [#Odor ‐ #Control] / [#Odor + #Control + #Other]^24^ (Figure 1B).

### 2.7 Confocal imaging

Before imaging, the worms were mounted on a 2% agar pad containing 5 mM NaN_3_ under a glass coverslip, and fluorescence was scored within 20 min to avoid the effects of NaN_3_. The quantification of the stress response evaluated by nuclear translocation TJ356 and EGL‐4 was performed using JZ500.

In the AWC neurons of each animal, EGL‐4 was localized to the cytoplasm or nucleus. In rare cases, when the site of the localization of the animal could be classified as nucleus or cytoplasm, we will calculate the worm as the nucleus and then calculate an extra animal. Next time this happens, we calculated the worm cytoplasm and an extra animal.^25^ Fluorescence values were quantified using the ImageJ software.

### 2.8 Gene expression analysis using real‐time PCR

Total RNA was extracted from approximately 1000 worms. The worms were starved again with the trained odorant for 2 h and then washed thrice in M9 and once in DEPC‐treated water. Total RNA was isolated from WT animals using RNA‐easy TM Isolation Reagent (Vazyme, China). Gene expression levels were normalized to *Act‐1* expression levels. RT‐PCR was carried out using random primers, the HiScriptⓇ II One Step qRT‐PCR SYBRⓇ Green Kit (Vazyme, China), and a real‐time PCR machine (Applied Biosystems QuantStudio Flex System). The primers for the genes were synthesized by Sangon Biotech (Shanghai, China), as follows: 5′‐TTCCAGTGATGGCTGACG‐3′ and 5′‐GGCTTCTAGGCCTACTTCG‐3′ for hsp‐12.6; 5′‐AGTGTGACTGCAAAAACAAGCAA‐3′ (forward) and 5′‐TCCACTGCATTCACATTTGTCTC‐3′ (reverse) mtl‐1; 5′‐CTGCGTAAACCTTCAAACTC‐3′ and5′‐ATGCGGAGTTACCATAGTTC‐3′ for cpr‐2; 5′‐CTAAGGATGGTGGAGAACCTTCA‐3′ and 5′‐CGCGCTTAATAGTGTCCATCAG‐3′ for sod‐3;and 5′‐ACCACGCTCGTATGCTGAAA‐3′ and 5′‐GTTTTCCTCCGCGTGGATTG‐3′ for rgs‐3; 5′‐GAGCACGGTATCGTCACCAA‐3′ (forward) and 5′‐TGTGATGCCAGATCTTCTCCAT‐3′ (reverse) for act‐1. act‐1 was used to normalize mRNA expression. The relative quantification of the gene expression was calculated by the 2^−ΔΔCT^ method.

### 2.9 Fitness (survival) analysis

Worms were starved again with the trained odorant for 2 h, transferred back to the food, and subjected to heat shock at 37°Cfor 4 h. The survival rate was counted the next day.

### 2.10 High‐performance liquid chromatography (HPLC)

About 1000–5000 juvenile wild type *C. elegans* were transferred to each NGM plate (90 mm diameter). Worms were harvested using cold M9 buffer after being treated with the 10 μM propofol and collected in 15 ml centrifuge tubes. The tubes were placed on ice for 10 min, then centrifuged for 2 min at 1150*g* to precipitate the worms. The worm pellets were rinsed three times with cold M9 buffer, freeze dry, and weighed. The worm pellets were resuspended in 1 ml methyl ethanol (HPLC grade) and sonicated. Then, the worm solution was centrifuged at 12,000*g* for 3 min. The propofol‐containing supernatant was transferred to a 1.5 ml centrifuge tube. Next, 10 ml of each sample was injected into the system and separated in a reversed‐phase C18 column (ZOBAX Eclipse XDB‐C18, 4.6 × 250 mm, 5 μm) containing 20% of CH_3_OH and 80% of H_2_O in the mobile phase, with a flow rate of 1.0 ml/min and a temperature of 40°C, and detected at 270 nm wavelength.

### 2.11 RNA interference (RNAi)

RNAi was induced by feeding as previously described. RNAi‐feeding constructs for L4440 and *rgs‐3* were generated by Suny Biotech (http://www.sunybiotech.com/). After exposure to propofol, L1 worms were then transferred to 6 × 6 cm plates and exposed to *E. coli* HT115 containing the expression plasmid for double‐stranded *rgs‐3* previously constructed^19^ at 20°C for approximately 48 h until the L4 stage corresponding to young adults was reached.

### 2.12 Statistical methods

The data were analyzed using GraphPad Prism 8. The normal distribution of continuous variables was tested using the Shapiro–Wilk test. Normally distributed variables are expressed as the mean (SD) and analyzed using an independent Student's *t*‐test or one‐way analysis of variance (ANOVA). Nonparametric variables are expressed as medians (interquartile range) and were compared using the Kolmogorov–Smirnov test or Kruskal–Wallis test. Differences were considered statistically significant at *p* < 0.05.

## 3 RESULTS

### 3.1 Propofol induces a reduction in learning capacity in a time‐ and dose‐dependent manner

Initially, N2 juvenile nematodes (L1 stage) were exposed to propofol (10 μM, 3 h) and observed at the L4 stage. Propofol decreased the percentage of nematodes who avoided IAA after training for 90 min (Figure 2B; Control vs. Propofol, MD [95% CI] = 30.17 [23.83 to 36.51], *p <* 0.001), indicating that exposure of juvenile *C. elegans* to propofol may alter the ability of learning.

The nematodes were then exposed to different concentrations of propofol for different durations. The chemotaxis index reflected the nematode preference for odor.

The results of the analysis showed a significant effect of propofol treatment [Figure 2C; *F*(4, 125) = 682.6, *p* < 0.001], time [*F*(4, 125) = 2296, *p* < 0.001] and propofol‐by‐time interaction [*F*(16, 125) = 113.5, *p* < 0.001]. This suggested that propofol caused learning to decrease in a time‐ and dose‐dependent manner.

**FIGURE 3:**
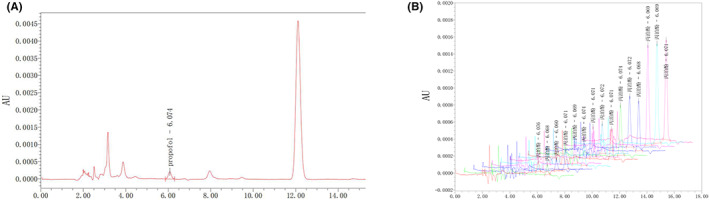
The HPLC profile of propofol. (A) The HPLC profile and retention time of propofol. (B) Comprehensive chromatogram of pending text sample and standard. The Chinese character in the picture means propofol). *n* = 3 independent experiments.

We then assessed the propofol concentration in nematodes by HPLC. To obtain the minimum concentration affecting the ability of aversive olfactory learning of adult nematodes, we set the propofol concentration at 10 μM. We exposed nematodes to propofol for 1, 3, and 10 h. To intuitively gauge the concentration of propofol in the nematode, we estimated the propofol concentration in the nematode effect chamber. We also estimated the propofol concentration in nematode neurons according to an 80% water content (Figure 3; Table 1).

**TABLE 1:** Concentration of propofol in *Caenorhabditis elegans* exposed to 10 μM propofol

Time	1 h	3 h	5 h
Concentration of propofol (μg/g)	3.872 ± 1.212	9.653 ± 1.755	28.205 ± 8.325
Effect chamber concentration (μg/ml)^a^	0.774 ± 0.242	1.931 ± 0.351	5.641 ± 1.665

*Note*: Data are expressed as mean ± SD.

^a^ The Effect chamber concentration of propofol was estimated as 80% of *C. elegans* moisture.

N2 juvenile nematodes (L1 stage) were exposed to propofol (10 μM, 3 h), and we observed whether nematodes lost the ability of learning by prolonging the training time. The results showed that with the extension of training time, the chemotaxis index decreased gradually [Figure 2D; *F*(1, 50) = 233.7, *p* < 0.001]. After the training time was extended to 12 h, no significant difference was observed between the propofol and control groups (Figure 2D; 12 h: control vs. propofol, MD [95% CI] = −0.05 [−0.13 to 0.03], *p* = 0.36; 24 h: control vs. propofol, MD [95% CI] = −0.01 [−0.09 to 0.07], *p* = 0.99). This suggests that propofol impairs the learning ability of nematodes by decreasing the speed of learning rather than by mediating a failure to learn in the worms.

### 3.2 Propofol induces a reduction in non‐associative and associative long‐term memory

**FIGURE 4:**
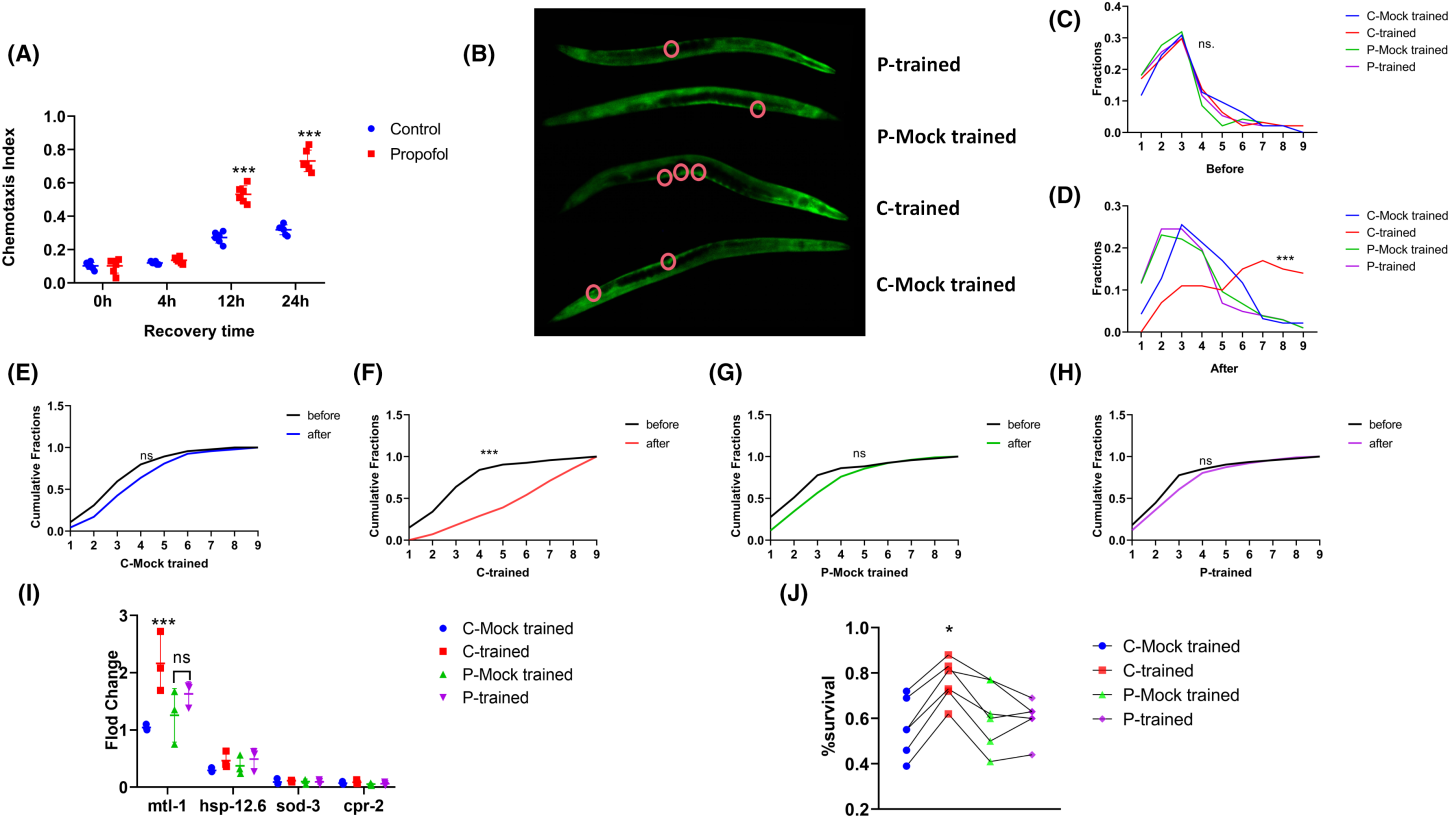
Propofol induces a decline in non‐associative and associative long‐term memory. (A) Chemotaxis index of nematodes exposed to IAA calculated at different recovery times. *n* = 40–60 animals per condition and the experiment was repeated six times. A one‐way analysis of variance (ANOVA) test was used. (B) Odor‐induced memory retrieval resulted in the rapid (within 20 min) translocation of DAF‐16 / FOXO to the nucleus (red circle) of gonadal sheath cells. To visualize protein spatial dynamics, we used a strain expressing translation fusion DAF‐16:: DAF‐16:: GFP (TJ356). Each of the four typical worms is imaged separately, cut along its edge, and stacked on top of the other. (C–D) Density plots of the number of cells with nuclear DAF‐16/FOXO localization per worm in different groups. *n* = ~100 animals per condition. A nonparametric Kruskal‐Wallis test was used. (E–H) Cumulative distribution of the number of cells with nuclear DAF‐16/FOXO localization per worm before or after the challenge. A nonparametric Kolmogorov–Smirnov test was used. (I) Expression of genes downstream of the DAF‐16/FOXO transcription factor was upregulated following odor‐induced memory reactivation. *n* = 3 independent experiments. One‐way analysis of variance (ANOVA) was used. (J) Each line represents a single independent experimental repeat (total *n* = 5), each with ~100 animals scored for each of the groups. Paired *t*‐test was used (*p < 0.05 versus other groups; ***p < 0.001 versus other groups). Before: before challenging; After: after challenging for 20 min.

N2 juvenile nematodes (L1 stage) were exposed to propofol (10 μM, 3 h), and the effect of propofol on the non‐associative memory of nematodes was observed. When the training duration was fixed at 24 h, with the extension of the recovery time, the chemotaxis index gradually increased [Figure 4A; *F*(1, 40) = 246.6, *p* < 0.001]. After 12 h of recovery, the chemotaxis index showed a statistically significant difference (Figure 4A; 12 h: control vs. propofol, MD [95% CI] = −0.26 [−0.31 to −0.20], *p* < 0.001; 24 h: control vs. propofol, MD [95% CI] = −0.41 [−0.47 to 0.36], *p* < 0.001). This showed that propofol led to a decline in long‐term memory, which was manifested in the acceleration in the speed of forgetting.

This study examined whether nematodes exposed to propofol at the L1 stage would demonstrate associative memory at the L4 stage. Here, we refer to the process of re‐exposing the worm to the odor as challenging the animal.

After training for 24 h and recovery for 4 h, C‐trained animals (Figure 1A) were exposed to IAA again, and DAF‐16/FOXO was induced to migrate rapidly (<20 min) to the nuclei. This rapid shift was primarily evident in the gonadal sheath cells. Although each gonad consisted of 10 sheath cells, the number of cells undergoing this rapid nuclear shift varied (Figure 4B). Therefore, we quantitatively analyzed the distribution of cells with nuclear DAF‐16/FOXO. The results showed that before the challenge with IAA, each group consisted of a small number of cells, typically 0–3 cells, with nuclear DAF‐16/FOXO, and no difference was observed in the number of cells with nuclear DAF‐16/FOXO (Figure 4C; K‐W = 2.86; *p* = 0.41). This showed that the stress level of nematodes in each group recovered to the same level observed after 24 h, and propofol did not alter the stress level of nematodes (Figure 4C; P‐mock trained median = 2, *n* = 94 compared with after median = 2, *n* = 94, K‐S = 0.04; *p* = 0.99). P‐trained worms did not show a significant increase in the number of cells with nuclear localization after re‐exposure to IAA rather than C‐trained worms (Figure 4D; P‐trained: before median = 3, n = 94 compared with after median = 3, *n* = 104, K‐S = 0.17; *p* = 0.12; C‐trained before median = 3, *n* = 94 compared with after median = 6, *n* = 100, K‐S = 0.55; *p* < 0.001).

We then focused on several known DAF‐16/FOXO‐dependent stress‐response genes, such as *cpr‐2*, *hsp‐12.6*, *mtl‐1*, and *sod‐3*, to further verify the stress response of nematodes. The expression level of *mtl‐1* increased significantly in C‐trained worms [Figure 4F; C‐mock‐trained vs. C‐trained; 1.04 (0.05) vs. 2.16 (0.52), *n* = 3, MD (95% CI) = −1.120 (−1.56 to −0.68); *p <* 0.001], while the expression level did not increase in P‐trained worms [Figure 4E; P‐mock‐trained vs. P‐trained; 1.257(0.47) vs.1.630(0.22), *n* = 3, MD (95% CI) = −0.37 (−0.81 to 0.07); *p* = 0.12]. No significant differences were observed in the expression of *cpr‐2*, *hsp‐12.6*, and *sod‐3* [Figure 4I; cpr‐2: *F*(3, 8) = 0.73, *p* = 0.56；hsp‐12.6: *F*(3, 8) = 1.10, *p* = 0.40; sod‐3: *F*(3, 8) = 0.19, *p* = 0.90].

A fitness (survival) analysis was used to confirm the implications of the stress response induced by the odor. The survival rate in heat sensitivity experiments can reflect the memory of nematodes related to odor and stress. When nematodes are exposed to the odor representing adversity again, the rapid stress response is conducive to their resistance to adversity, such as the high temperature stress in this experiment. Therefore, we believe that nematodes that successfully form associative long‐term memory can better resist the stress of high temperature. The results showed that the survival rate of C‐trained animals was high [Figure 4J; C‐Mocked trained vs. C‐trained; *n* = 5, *t* = 9.65; *p* < 0.001]; however, P‐trained animals had a low survival rate [Figure 4J; P‐mock‐trained vs. P‐trained; *n* = 5, *t* = 0.20, *p* = 0.85; P‐ trained vs. C‐trained; *n* = 5, *t* = 12.3, *p* < 0.001]. Therefore, we believe that propofol leads to the impairment of associative long‐term memory.

### 3.3 Propofol induced upregulation in the expression of *rgs‐3* and a decline in the ability of nuclear accumulation of EGL‐4/PKG in AWC neurons

**FIGURE 5:**
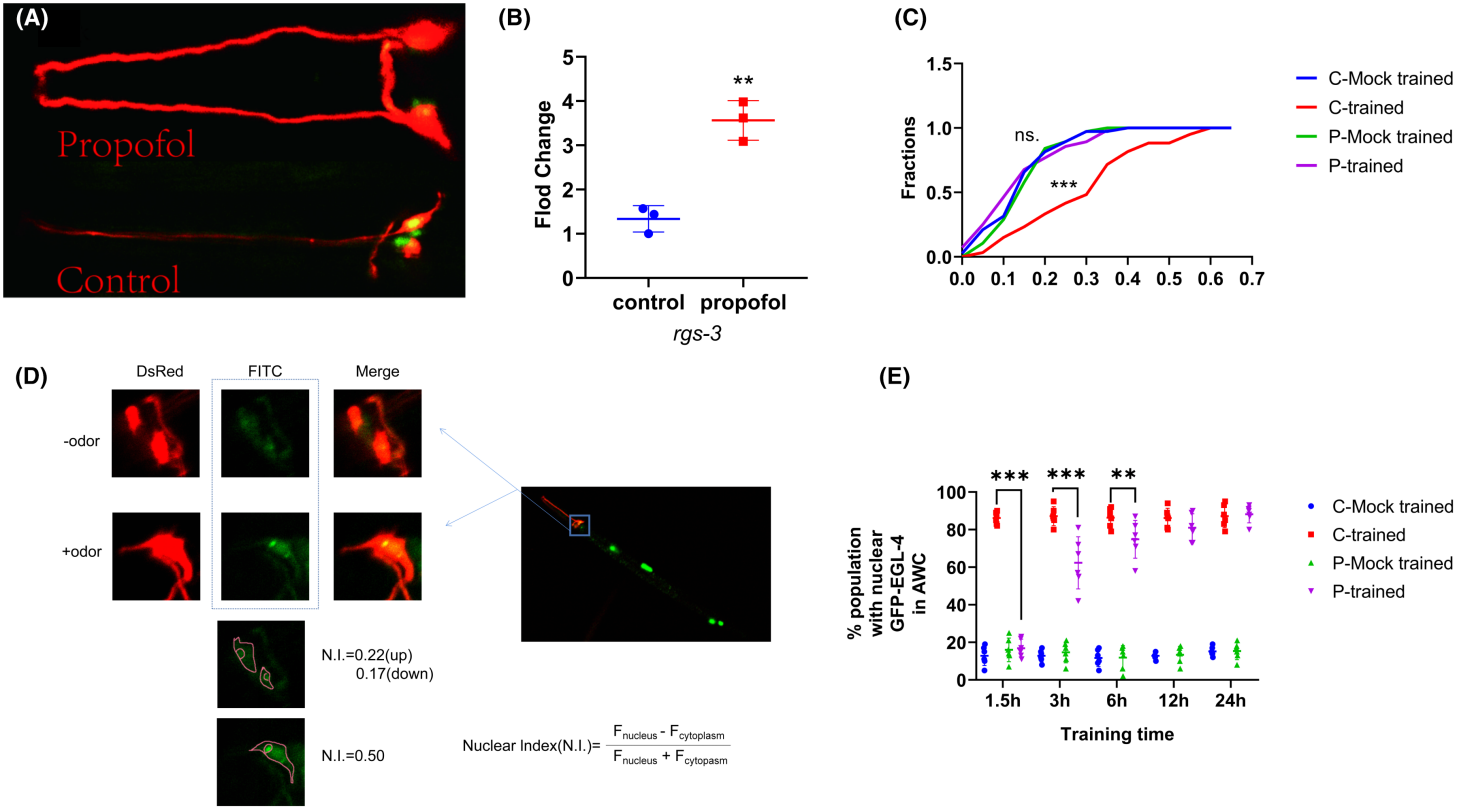
Propofol induces upregulation of the expression of rgs‐3 and a decline in the ability of nuclear accumulation of EGL‐4/PKG in AWC neurons. (A) Fluorescent confocal images of AWC neurons in propofol and control groups. Integrality of the cilia of AWC neurons was observed in the propofol and control groups. Each of the two typical worms is imaged separately, cut along its edge, and stacked on top of the other. (B) The expression of *rgs‐3* was up‐regulated after exposure to propofol at the L4 stage. *n* = 3 independent experiments. The *t*‐test was used. (C) Cumulative distribution (AWC neuronal nuclear index) for EGL‐4::GFP in different groups of animals after conditioning. An increase in AWC neuronal nuclear index was observed after conditioning in the C‐trained group, while a decrease was observed after conditioning in the P‐trained group. *n* = 40–60 animals per condition. A nonparametric Kruskal‐Wallis test was used. (D)Nuclear enrichment of EGL‐4 in AWC neurons. Representative images of EGL‐4::GFP fluorescence in the AWC neurons. *F*_nucleus_, *F*_cytoplasm_ = fluorescence measured in AWC neuron (nucleus or cytoplasm of the same neuron). (E) Percentage of animals with nuclear EGL‐4 at different training intervals. *n* = 40–60 animals per condition, and the experiments were repeated six times. One‐way analysis of variance (ANOVA) was used (***p < 0.001; **p < 0.01).

We observed the morphology of AWC neurons. We did not observe any apparent damage induced by propofol (10 μM, 3 h, L1 stage) to the cilia of AWC neurons (Figure 5A).

The transcriptional levels of *rgs‐3* were measured before training (L4 stage). The results showed that the expression of *rgs‐3* was upregulated after exposure to propofol at the L4 stage, and the difference was statistically significant [Figure 5B; control vs. propofol: 1.15 (0.25) vs.3.56 (0.45), *n* = 3, *t* = 7.17; *p* = 0.002].

Quantitative microscopic analysis of the EGL‐4::GFP transgene expressed in AWC neurons was performed to determine the cytoplasmic and nuclear levels of EGL‐4 after exposure to odor and food deprivation simultaneously (Figure 5C). Odor regulation resulted in EGL‐4:: GFP enrichment in the nuclei of AWC neuron within 90 min in C‐trained animals (Figure 5D; C‐mock‐trained median = 0.15, *n* = 38 compared with C‐trained median = 0.32, *n* = 60, MD = −54.33; *p* < 0.001). However, the nematodes exposed to propofol in the L1 stage demonstrated a failure of re‐localization of EGL‐4::GFP in AWC neurons after aversive olfactory training (Figure 5D; P‐mock‐ trained median = 0.15, *n* = 38 compared with P‐trained median = 0.13, *n* = 56, MD = 11.70; *p* > 0.99).

The percentage of nematodes showing nuclear translocation of EGL‐4 was determined. The results also showed that with the extension of training time, the number of EGL‐4 nuclear translocations increased gradually in P‐trained worms [Figure 5E; *F*(3, 100) = 1106, *p* < 0.001]. After the training time was extended to 12 h, no significant differences were observed between the P‐trained and C‐trained groups (Figure 5E; 12 h:C‐trained vs. P‐trained, MD [95% CI] = 5.17 [−4.00 to 14.33], *p* = 0.46; 24 h:C‐trained vs. P‐trained, MD [95% CI] = −1.00 [−10.17 to 8.17], *p* = 0.99). This suggests that propofol does not induce any permanent damage to the nuclear translocation of EGL‐4:: GFP.

### 3.4 Inhibiting the expression of *rgs‐3* could alleviate propofol‐induced reduction in the memory and the ability to learn

**FIGURE 6:**
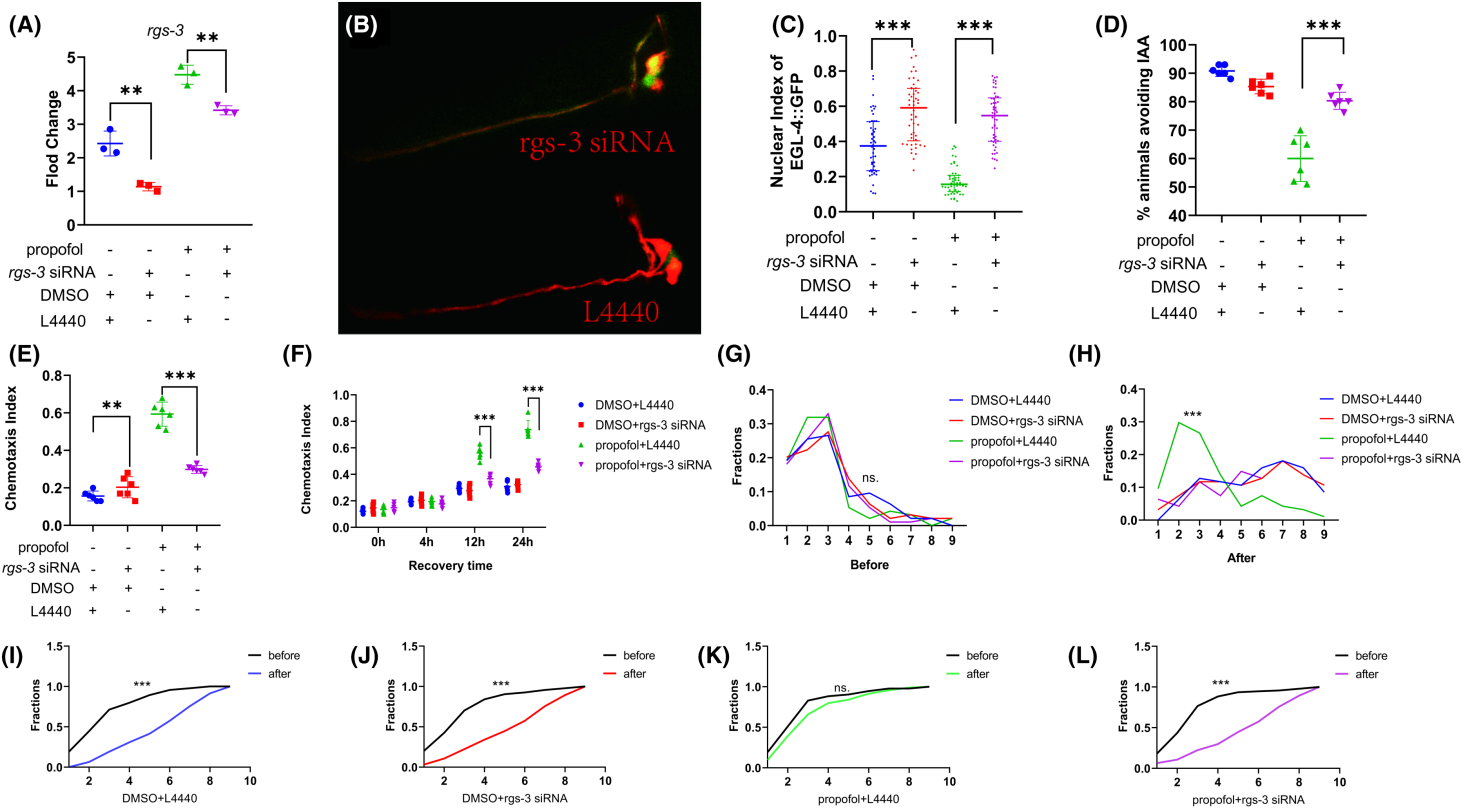
Inhibition of the expression of *rgs‐3* could alleviate the propofol‐induced decline in learning and memory. (A) The expression of *rgs‐3* was down‐regulated after feeding RNAi. *n* = 3 independent experiments. One‐way analysis of variance (ANOVA) was used. (B) Fluorescent confocal images of AWC neurons in *rgs‐3* siRNA and L4440. The integrality of the cilia of AWC neurons was observed. Each of the two typical worms is imaged separately, cut along its edge, and stacked on top of the other. (C) AWC neuronal nuclear index for EGL‐4::GFP in different groups of animals after conditioning. The bar represents the median and interquartile range. *n* = 40–60 animals per condition. A nonparametric Kruskal–Wallis test was used. (D) Percentage of animals avoiding IAA after feeding RNAi. One‐way analysis of variance (ANOVA) was used. (E) Chemotaxis index of nematodes exposed to IAA at 1.5 h of training interval. One‐way analysis of variance (ANOVA) was used. (F) Chemotaxis index of nematodes exposed to IAA at different recovery times. *n* = 40–60 animals per condition and repeated thrice. A one‐way analysis of variance (ANOVA) test was used. (G‐H) Density plots of the number of cells with nuclear DAF‐16/FOXO localization per worm in different groups. *n* = ~100 animals per condition. A nonparametric Kruskal–Wallis test was used. (I–L) Cumulative distribution of the number of cells with nuclear DAF‐16/FOXO localization per worm before or after the test in P‐trained worms. A nonparametric Kolmogorov–Smirnov test was used (***p < 0.001; **p < 0.01).

In this study, nematodes were fed with E. coli expressing dsRNA during the late L1 stage. The expression of *rgs‐3* was downregulated before the worms were trained [Figure 6A; DMSO+L4440 vs. DMSO+*rgs‐3* siRNA; 2.43 (0.37) vs. 1.14 (0.13), *n* = 3, MD (95% CI) = 1.29 (0.63 to 1.94); *p* = 0.001; propofol+L4440 vs. propofol+*rgs‐3* siRNA; 4.48 (0.28) vs. 3.42 (0.13), *n* = 3, MD (95% CI) = 1.06 (0.40 to 1.72); *p* = 0.003].

We performed RNA interference with JZ500 to observe the morphology of AWC neurons and nuclear accumulation of EGL‐4. We did not find that RNAi induced any apparent damage to the cilia of AWC neurons (Figure 6B). The finding is in contrast to the previously reported cilial damage observed in AWC neurons in the *rgs‐3* mutant.^21^

The nematodes who were exposed to propofol (10 μM, 3 h, L1 stage) and demonstrated inhibited *rgs‐3* expression began to re‐localize EGL‐4::GFP in AWC neurons after aversive olfactory training (Figure 6C; propofol+L4440 median = 0.16, *n* = 50 compared with propofol+*rgs‐3* siRNA median = 0.55, *n* = 50, MD = −101.2; *p* < 0.001), and the percentage of worms who avoided IAA source began increasing [Figure 6D; propofol+L4440 vs. propofol+*rgs‐3* siRNA; 60.00 (8.03) vs.80.33 (0.13), *n* = 6, MD (95% CI) = −20.33 (−27.73 to −12.93); *p* < 0.001], and the chemotaxis index reduced [Figure 6E; propofol+L4440 vs. propofol+*rgs‐3* siRNA; 0.59 (0.06) vs.0.30 (0.02), *n* = 6, MD (95% CI) = 0.30 (0.22 to 0.40); *p* < 0.001]. These findings imply that inhibiting the expression of *rgs‐3* could alleviate propofol‐induced reduction in the ability to learn.

After 12 h of recovery, the chemotaxis index of nematodes exposed to propofol and those with inhibited *rgs‐3* expression decreased [Figure 6F; 12 h: propofol+L4440 vs. propofol+*rgs‐3* siRNA; 0.56 (0.05) vs.0.37 (0.04), *n* = 6, MD (95% CI) = 0.20 (0.14 to 0.25); *p* < 0.001; 24 h: propofol+L4440 vs. propofol+*rgs‐3* siRNA; 0.74 (0.07) vs.0.46 (0.03), *n* = 6, MD (95% CI) = 0.28 (0.23 to 0.33); *p* < 0.001]. The number of cells showing rapid nuclear translocations of DAF‐16/FOXO increased in nematodes exposed to propofol and *rgs‐3* siRNA (Figure 6H; propofol+*rgs‐3* siRNA: the median before exposure =3, *n* = 94; the median after exposure = 6, *n* = 94, K‐S = 0.59; *p* < 0.001). This finding showed that inhibiting the expression of *rgs‐3* could alleviate propofol‐induced memory deficits.

## 4 DISCUSSION

Studies have shown that *C. elegans* can associate long‐term memories. When the nematode has experienced long‐term adversity, such as hunger, in an environment containing a specific odor molecule, upon repeated exposure to this odor, the nematode produces corresponding endogenous stress changes to resist the upcoming adversity.^26^ However, this is the first study to use *C. elegans* as a model organism to show that anesthetic exposure to neonatal neurons results in a decrease in learning and memory.

As a model organism, *C. elegans* has many advantages over rodents in terms of the examination of learning and memory deficits induced by chemical exposure during neurodevelopment. First, the feeding cycle of nematodes is short, which reduces the time and capital costs of the experiment.^27^ Second, in the process of detecting learning and memory function, the experimental conditions of the nematode are more stable, with fewer interference factors. Hence, the experimental results of studies examining learning and memory function in *C. elegans* are more reliable and reproducible.^28^ Third, the nematode lacks a cardiovascular system, and learning and memory function changes cannot be related to perturbations in cerebral perfusion, which might be implicated in mammals.^7^ Finally, the genetic and optical properties of nematodes render the study of related mechanisms easier.^29^ Therefore, nematodes are advantageous to a certain extent in studying the mechanism and observing the phenomenon in vitro and in vivo.

However, there are still many differences between *C. elegans* and mammals. For example, in terms of gender, most *C. elegans* are hermaphroditic, and few are male individuals. We cannot observe the effect of gender on cognition in worms, although in mammals, studies have found that gender has a greater impact on cognitive function.^30^, ^31^ Therefore, we believe that the *C. elegans* experiment cannot completely replace the mammalian experiment.^32^ However, nematodes can be used as a bridge between cell experiments and animal experiments to verify the results of cell experiments and avoid the failure of animal experiments as much as possible. Considering that people who study the neurotoxicity of anesthetics seem to have neglected nematodes as a good model animal.^33^ This study reported how to observe the cognitive function of nematodes according to their behavioral changes.

This study demonstrated the phenomenon of the damage of learning and memory induced by exposure to propofol in juvenile *C. elegans* from three perspectives: (1) the damage was induced by propofol in a time‐ and dose‐dependent manner; (2) the damage involved the decline in learning speed and the acceleration of the speed of forgetfulness; and (3) associative long‐term memory was impaired.

In the natural state, nematodes are attracted by IAA odor molecules; therefore, the chemotaxis index of nematodes to IAA is close to one. However, when nematodes received aversive olfactory training for 90 min, they dislike IAA odor; therefore, the chemotaxis index will be reduced. Therefore, we were able to examine the learning ability of nematodes by detecting their chemotaxis index after training.

At present, no studies have been published examining the effects of intravenous anesthetics on nematodes. In this study, propofol, an intravenous anesthetic, was studied as a chemical with neurotoxic potential for the developing nervous system. It cannot be confirmed whether propofol has the same anesthetic effect on nematodes as that observed with anesthetics administered via inhalation.

Nevertheless, propofol reportedly damages the developing brain in mammals. We believe that the nematode model is a good supplement to these studies. First, in most studies, researchers usually observe the damaging effect of anesthetics on neuronal cells; however, neuronal damage in vitro does not necessarily reflect behavioral changes in vivo. Therefore, part of in vitro experiments cannot be verified in animals. We believe it is efficient and scientifically sound to use nematodes to demonstrate the conclusions of cell experiments before moving forward with animal experiments. Second, in this type of study, the dosage of narcotic drugs is often much higher than the clinical dosage. This study confirmed through HPLC that propofol, even if at a concentration far lower than the clinical dose, still has a long‐term damaging effect on nematode learning and memory abilities, which differs from previous studies.

After elucidating the experimental phenomena, the experimental mechanism was studied. RGS proteins potently modulate the function of heterotrimeric G‐proteins by stimulating GTPase activity of G‐protein alpha subunits.^34^ Considering the important role of G‐proteins in synaptic plasticity, it is not difficult to understand the impact of RGS on learning and memory.^35^ Rodent studies have shown that RGS mRNA is specifically expressed in different brain regions^36^ and that RGS14 may integrate these diverse signaling pathways to modulate synaptic plasticity in CA2 hippocampal neurons.^37^ In *C. elegans*, although no direct evidence is available showing the effect of RGS on learning and memory, the important role of *egl‐4*/cGMP‐dependent protein kinase G (PKG) in the learning process and the effect of the Gαq protein on memory prompted us to hypothesize that RGS still plays an important role in learning and memory. Many factors can lead to changes in RGS mRNA expression in the rodent brain, including acute stress and drug exposure.^38^ RGS has been widely studied in drug addiction.^13^ However, no study has reported the effect of propofol on RGS, despite the fact that propofol is also addictive. This study is the first to report that propofol can alter the expression of RGS and RGS plays an important role in learning and memory in *C. elegans*.

EGL‐4 is homologous to PKG, which is closely associated with olfactory learning.^22^, ^24^, ^25^ *egl‐4* mutants show a learning deficit in the perception of multiple odors in AWC neurons.^21^ EGL‐4 is transferred from the cytoplasm to the nucleus in AWC neurons, phosphorylating the heterochromatin protein HPL‐2 and altering gene expression during odor conditioning.^24^ The nuclear translocation of EGL‐4 is a real‐time marker of AWC neuronal plasticity.^25^ The results showed that exposure to propofol affects the nuclear translocation of EGL‐4 in AWC neurons. This may be associated with the increased expression of RGS‐3.

Therefore, we used RNA interference to disrupt the mRNA expression of *rgs‐3* in *C. elegans* after exposure to propofol. Considering the damage to chemotaxis in the *rgs‐3* mutant and the damage to neuronal cilia in AWC neurons,^21^ we did not use the *rgs‐3* mutant and did not select nematodes with a stable phenotype after RNA interference. The PCR results showed that the expression of *rgs‐3* was significantly downregulated in *C. elegans* after RNA interference before training. Simultaneously, we found that inhibiting the high expression of *rgs‐3* alleviated propofol‐induced decline in EGL‐4 nuclear translocation and improved learning and memory at the L4 stage.

RGS proteins are subject to many post‐translational modifications such as phosphorylation, palmitoylation, and sulfonylation. There are no consistent effects of phosphorylation on RGS proteins.^39^ Then, whether phosphorylation of rgs‐3 can affect neuronal damage may be a possible research direction. The RhoGEF family of RGS proteins functions as a GTPase‐activating protein (GAP) and an effector for Gα12/13. RGS‐RhoGEFs, including p115RhoGEF, leukemia‐associated RhoGEF (LARG), and PDZ‐RhoGEF, provide a direct link between Gα12/13‐coupled GPCRs and Rho activation. Furthermore, Rho activation is required for semaphorin‐induced growth cone collapse in neuronal cells, possibly explaining the possible downstream mechanisms of the propofol‐induced neuronal damage. This direction should be validated at the endogenous expression levels of RGS proteins in the future.^40^

RGS12 can interact with some receptor tyrosine kinases (RTK) like the nerve growth factor (NGF) receptor tyrosine kinase TrkA. Furthermore, RGS12 facilitated ERK activation by PDGF in CHO‐K1 cells and by NGF in PC12 cells. Besides, RGS12 was required for NGF‐mediated neurite outgrowth of PC12 cells.^41^ The growth of neurites may play a role in learning and memory development.

This study has some limitations. For instance, the mechanism underlying the induction of high *rgs‐3* expression sustained by propofol remains unelucidated. Moreover, although our study showed that *rgs‐3* may have an impact on learning and memory, it is unclear whether *rgs‐3* mediates its effects via the G protein and EGL‐4. This facet will be examined in our next studies. However, it is important to verify the effect of propofol on RGS and whether regulation of RGS expression can alleviate the neurotoxicity induced by propofol in mammals.

In conclusion, our findings show that the exposure of L1 *C. elegans* to propofol resulted in the upregulation of the expression of *rgs‐3* and failure of EGL‐4 nuclear translocation. These changes may lead to a decline in learning and memory. Inhibition of the expression of *rgs‐3* alleviated propofol‐induced learning and memory deficits.

## AUTHOR CONTRIBUTIONS

Study design: A.Z., S.Q.; Writing up of the first draft of the manuscript: A.Z., S.Q.; Manuscript editing: S.Q., A.Z., H.J., G.F., L.Y., C.L., Q.L., X.M., T.Z., S.L., Y.G.; Exposed propofol to worms: Q.L., S.L.; Acquisition of behavioral data: H.J., X.M.; Analysis of behavioral data: H.J., X.M., Q.L.; Observation of worms under confocal microscope: H.J., X.M., G.F.; Counts of nuclear translocation of EGL‐4::GFP and DAF‐16/FOXO: H.J., X.M.; Quantitative microscopic analysis: A.Z.; RT‐PCR: H.J.; RNAi feeding: H.J., G.F.; High‐performance liquid chromatography (HPLC): H.J., Y.G.; Statistical analysis of all data: C.L., A.Z., Y.L.

## CONFLICT OF INTEREST

The authors declare no conflicting financial interest.

## Data Availability

All data that support the findings of the current study are available from the corresponding authors upon reasonable request.
